# The Australian Public is Still Vulnerable to Emerging Virulent Strains of West Nile Virus

**DOI:** 10.3389/fpubh.2014.00146

**Published:** 2014-09-17

**Authors:** Natalie A. Prow, Elise K. Hewlett, Helen M. Faddy, Flaminia Coiacetto, Wenqi Wang, Tarnya Cox, Roy A. Hall, Helle Bielefeldt-Ohmann

**Affiliations:** ^1^School of Chemistry and Molecular Biosciences, The University of Queensland, St Lucia, QLD, Australia; ^2^Australian Infectious Diseases Research Centre, The University of Queensland, St Lucia, QLD, Australia; ^3^Research and Development, Australian Red Cross Blood Service, Kelvin Grove, QLD, Australia; ^4^School of Veterinary Science, The University of Queensland, Gatton, QLD, Australia; ^5^Vertebrate Pest Research Unit, NSW Department of Primary Industries, Orange, NSW, Australia; ^6^Invasive Animals Cooperative Research Centre, University of Canberra, Bruce, ACT, Australia

**Keywords:** West Nile virus, equine encephalitis, seroprevalence, humans, rabbits

## Abstract

The mosquito-borne West Nile virus (WNV) is responsible for outbreaks of viral encephalitis in humans and horses with particularly virulent strains causing recent outbreaks in Eastern Europe, the Middle East, and North America. In Australia, a strain of WNV, Kunjin (WNV_KUN_), is endemic in the north and infection with this virus is generally asymptomatic. However, in early 2011, following extensive flooding, an unprecedented outbreak of WNV_KUN_ encephalitis in horses occurred in South-Eastern Australia, resulting in more than 1,000 cases and a mortality of 10–15%. Despite widespread evidence of equine infections, there was only a single mild human case reported during this outbreak. To understand why clinical disease was seen in horses without similar observations in the human population, a serosurvey was conducted using blood donor samples from areas where equine cases were reported to assess level of flavivirus exposure. The seroprevalence to WNV_KUN_ in humans was low before the outbreak (0.7%), and no significant increase was demonstrated after the outbreak period (0.6%). Due to unusual epidemiological features during this outbreak, a serosurvey was also conducted in rabbits, a potential reservoir host. Out of 675 animals, sampled across Australia between April 2011 and November 2012, 86 (12.7%) were seropositive for WNV_KUN_, with the highest prevalence during February of 2012 (28/145; 19.3%). As this is the first serological survey for WNV_KUN_ in Australian feral rabbits, it remains to be determined whether wild rabbits are able to develop a high enough viremia to actively participate in WNV transmission in Australia. However, they may constitute a sentinel species for arbovirus activity, and this is the focus of on-going studies. Collectively, this study provides little evidence of human exposure to WNV_KUN_ during the 2011 outbreak and indicates that the Australian population remains susceptible to the emergence of virulent strains of WNV.

## Introduction

Flaviviruses are a group of medically important arboviruses causing large disease outbreaks around the world with approximately 50 million cases per year. Mosquito-borne flaviviruses in the Japanese encephalitis virus (JEV) serogroup, including JEV, West Nile virus (WNV), and Murray Valley encephalitis virus (MVEV), cause severe, potentially fatal neurological disease in humans, horses, and some avian species. WNV has traditionally been associated with outbreaks of viral encephalitis in Europe and Africa (1). In 1999, WNV appeared for the first time in the USA, associated with an outbreak of a fatal or debilitating disease in humans and equines, and extremely high levels of morbidity and mortality in several species of native birds in New York (2, 3). Since its introduction into the USA, WNV has caused more than 16,196 human cases of neuroinvasive disease and more than 1549 deaths in the USA alone and spread to most parts of North, Central, and South America via mosquito-bird transmission cycles (4).

The Kunjin strain of WNV (WNV_KUN_) is a closely related virus from Australia. Although WNV_KUN_ was initially considered a separate species in the flavivirus genus, studies by our laboratory and collaborators revealed that it shared a high degree of antigenic and genetic homology to WNV strains, justifying re-classification of the virus as a subtype of WNV (5–7). Until recently, the relatively benign WNV_KUN_, had only been associated with a few cases of non-fatal encephalitis in humans and a small number of equine cases since it was first isolated in 1960 (8). However, in early 2011 an unprecedented outbreak of equine encephalitis occurred in South-East Australia, causing mortality of 10–15% of horses infected (9). Upon isolation, a new equine virulent strain of WNV_KUN_ was confirmed, being the first strain to cause a major outbreak in Australia (9). Symptoms of equine infection with this strain included ataxia, muscle paralysis and tremors, changes in temperament, incoordination, and general weakness, which are consistent with clinical signs of the equine WNV encephalitis caused by virulent North American (WNV_NY99_) and European strains (10, 11). Notably, this new WNV strain, named WNV_NSW2011_, arose in regions of Southern Australia where WNV_KUNV_ had not been observed previously, including coastal and inland cities of New South Wales (NSW) (9).

Abundant rainfall in the latter half of 2010 and extending into the first quarter of 2011 led to extensive flooding of inland areas of NSW. This unforeseen event provided ideal climatic conditions for breeding of freshwater mosquito populations, which recorded a sixfold increase in number compared to the previous season (12). This prolific population growth of the primary vector for WNV_KUN_ is presumed to have spurred the major outbreak in 2011 (13, 14). Nevertheless, only a small number of human infections were recorded during the time period of the equine encephalitis epidemic. Perhaps even more curiously, a large number of the equine cases occurred east of the Great Dividing Range in the much dryer coastal regions where no flooding was experienced during the same period and mosquito populations were low (14). This prompted us to consider epidemiological factors other than a bird–mosquito–human transmission chain. One animal species that occurs in abundance in the main affected regions is the rabbit (*Oryctolagus cuniculus*). Feral rabbits have previously been shown to sustain a brief viremia sufficient to transmit to mosquitoes when experimentally infected with MVEV (15). Similarly, Eastern cottontail rabbits (*Sylvilagus floridanus*) develop a viremia sufficient for mosquito transmission when experimentally infected with WNV_NY99_ (16), and preliminary work in our group had shown that domestic rabbits (*O. cuniculus*) can become infected with WNV_NSW2011_ (17).

We therefore carried out a serological survey on humans from east-coast regions of NSW with high incidences of equine encephalitis cases, using plasma samples obtained from the Australian Red Cross Blood Service (Blood Service), to assess human exposure during the outbreak and evaluate the on-going risk of virulent strains of WNV_KUN_. In addition, we tested feral rabbits sampled by the NSW Department of Primary Industries (NSW DPI) from two areas in NSW and one area in all other states during and up to 1.5 years after the equine epidemic. Collectively, our data suggest that an overwhelming proportion of the Australian human population remains susceptible to the emergence of virulent strains of WNV, and that feral rabbits may represent a possible reservoir, at least in some areas of South-Eastern Australia.

## Materials and Methods

### Human plasma samples

**Figure 1:**
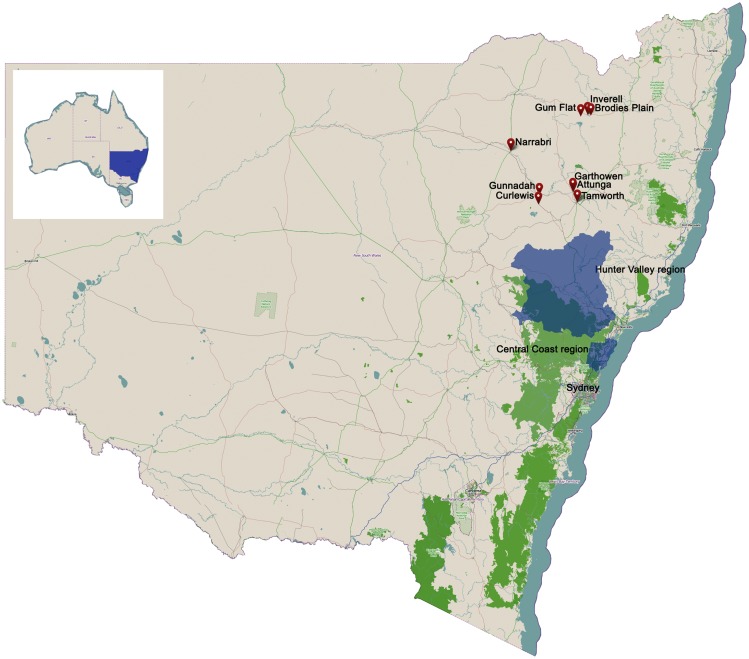
**Location of sampling areas for human blood donors in NSW, Australia**.

Plasma samples were acquired from healthy Australian blood donors after routine infectious disease testing was complete. Samples were selected from donors residing in eastern NSW coinciding with locations of WNV_NSW2011_ equine infection [Figure 1; see also Figure 3 in Ref. (14)], with samples collected from November 2009 to November 2010 (*n* = 148) forming the pre-2011 sample group and those from September 2013 (*n* = 168) forming the post-2011 sample group. Samples in the pre-2011 group were collected into EDTA plasma preparation tubes [Becton, Dickson and Company (BD) Biosciences, San Diego, USA], while samples collected in the post-2011 group were collected into EDTA tubes (BD Vaccutainer^®^ Whole Blood Collection tube). All samples were centrifuged and plasma aliquots archived at −30°C. Donor demographic data were obtained for each sample (age, gender, suburb, and postcode). This study was carried out under approval from the Blood Service Human Research Ethics Committee.

### Rabbit serum samples

**Figure 2:**
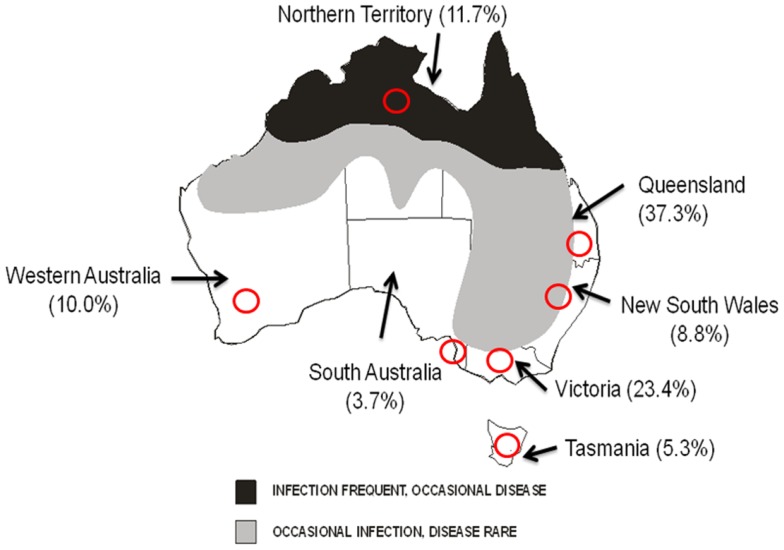
**Known distribution of WNV_KUN_ in Australia and seroprevalence in Australian rabbits**. States and Territories are shown. Red circles indicate approximate areas of rabbit sampling. Numbers in brackets represents overall seroprevalence of antibodies to WNV_KUN_ in rabbits during 2011–2012.

Serum samples derived from rabbits captured or shot as part of surveys conducted by NSW DPI in all states of Australia were made available for testing. Collections were performed in two central areas of NSW (Euchareena and Oaky Creek in the central tablelands), and one area in each of the other states and the Northern Territory (near Hattah-Kulkyne National Park in Victoria; near Coorong in South Australia; south-east Queensland; South-West Western Australia; Figure 2) during seven time periods between April 2011 (Autumn) and November 2012 (Spring). A total of 675 samples were available for testing. The age of the rabbits was determined by lens dry weight measurements as described previously (18).

### Epitope blocking ELISA

Virus-specific antibodies to WNV_KUN_ or MVEV were detected in human plasma and rabbit sera using a blocking ELISA (bELISA) (19) as detailed in Prow et al. (20). Seropositivity is defined as inhibition of the binding of virus-specific monoclonal antibodies (mAb) by more than 30%. All samples were initially screened for flavivirus antibodies using the anti-flaviviral E protein mAb 4G2 as the competing antibody (20). All flavivirus positive samples were then tested for WNV_KUN_ (both human and rabbit samples) and MVEV (human samples only) using the same bELISA protocols with the mAbs 3.1112G and 10C6, respectively. Horse sera previously tested to be positive and negative for the virus of interest (20) were employed as controls for the bELISA on human plasma, while serum from naïve SPF-bred New Zealand White rabbits and rabbits experimentally infected with WNV_NSW2011_ (kindly provided by W. Suen, University of Queensland) were used as negative and positive controls for the bELISA involving rabbit sera. Controls consisting of no sera and no antigen (coating buffer only) were also included in each bELISA.

### Virus neutralization assay

Samples positive in the bELISA were tested for neutralizing activity against WNV_KUN_ (rabbit and human samples) and MVEV (human samples only) in a microtiter serum neutralization assays, using MVEV (strain 1–51) or WNV_KUN_ (strain MRM16) as previously described in detail (20).

### Statistical analysis

The proportion seropositive and 95% CI was calculated. For both human and rabbit data, seropositivity was first compared across age group, location (state/region), sex (human only), and time period in univariate analyses using the chi-squared test or a univariate logistic regression. Where a significant relationship with seropositivity was observed for two or more variables, these variables were entered into a logistic regression model [with seropositivity (reactive or non-reactive) as the dependent variable (non-reactive as the reference) and the other variables as factors]. Microsoft Excel (Microsoft Pty. Ltd., North Ryde, NSW, Australia) and the Statistical Package for the Social Sciences (SPSS; IBM Australia Ltd., St. Leonards, NSW, Australia) were used for data management and analyses.

## Results

### Profile of the human study populations

A total of 316 individuals were included in this study, consisting of 148 in the pre-2011 cohort and 168 in the post-2011 cohort (Table 1). Just over half (52.2%) of the samples were from male donors. The median age of donors was 43 years (IQR 26–56) for the pre-2011 sample group and 51 years (IQR 40.5–61) for the post-2011 sample group, with a slightly skewed distribution toward older donors in the post-2011 sample group. The majority of samples were collected from regional NSW, west of the Great Dividing Range (Table 1).

**Table 1:** **Characteristics of the human study population**.

Time point/region	Number of samples	Age group						Male (%)
		≤24	25–34	35–44	45–54	55–64	≥65	
**PRE-2011**								
Hunter valley/Central Coast/Sydney	16	8	1	2	1	3	1	38
Regional NSW	132	26	20	23	21	22	20	52
Total	148	34	21	25	22	25	21	50
**POST-2011**								
Hunter valley/Central Coast/Sydney	32	4	1	5	11	8	3	59
Regional NSW	136	16	11	20	31	35	23	53
Total	168	20	12	25	42	43	26	54
**TOTAL**								
Hunter valley/Central Coast/Sydney	48	12	2	7	12	11	4	52
Regional NSW	268	42	31	43	52	57	43	52
Total	316	54	33	50	64	68	47	52

### Seroprevalence of flaviviruses, WNV_KUN_, and MVEV in human beings in coastal NSW

Flavivirus total antibody was detected in 15 of the 148 samples in the pre-2011 group (10.1%), while 13 of 168 (7.7%) samples were observed to be seropositive for flavivirus antibody in the post-2011 group (Table 2). Seroprevalence of WNV_KUN_ total antibody in the pre-2011 samples was 0.7% with just one sample testing seropositive, which is similar to the seroprevalence observed in the post-2011 samples (0.6%) where again, just one sample was observed to be seropositive to WNV_KUN_ (Table 2). These two WNV_KUN_ seropositive samples were both from males, with ages of 68 and 65, respectively, both of whom were residents of Tamworth in the North West Slopes subregion of regional NSW. Both WNV_KUN_ seropositive samples were also tested in the neutralization assay and confirmed to have neutralizing antibodies against WNV_KUN_ with titers of 40 and 80, respectively. None of the total flavivirus seropositive samples tested positive for antibodies against MVEV.

**Table 2:** **Flavivirus seroprevalence in blood donors from eastern NSW collected in the months prior to and soon after the equine 2011 WNVKUN epidemic**.

Time point	Number tested	Total flavivirus		WNV_KUN_	
		Positive	% (95% CI)	Positive	% (95% CI)
Pre-2011	148	15	10.1 (5.27–15.00)	1	0.7 (0.00–2.00)
Post-2011	168	13	7.8 (3.70–11.78)	1	0.6 (0.00–1.76)
Overall	316	28	8.9 (5.73–11.99)	2	0.6 (0.00–1.51)

No significant difference was observed between pre- and post-2011 time points in either total flavivirus seropositivity (*p* = 0.454) or in WNV_KUN_ seropositivity (*p* = 0.928). Overall, the proportion of males seropositive for total flavivirus antibody was significantly higher than females (*p* = 0.001); however, age group (*p* = 0.146) and region (*p* = 0.889) did not significantly influence seropositivity (Table 3).

**Table 3:** **Breakdown of total flavivirus seropositivity by sex, age group, and region**.

Variable	Number tested	Total flavivirus seropositivity		Univariate analysis	
		*n*	% (95% CI)	Odds ratio (95% CI)	*p*-Value
**Time period**
Pre-2011	148	15	10.1 (5.27–15.00)	†	–
Post-2011	168	13	7.8 (3.70–11.78)	1.345 (0.618–2.928)	0.456
**Sex**
Female	151	5	3.3 (0.46–6.17)	†	–
Male	165	23	13.9 (8.65–19.22)	4.730 (1.750–12.784)	0.002
**Age group (years)**					**0.242**
<25	54	1	1.8 (0.00–5.45)	†	–
25–34	33	3	9.1 (0.00–18.90)	5.30 (0.528–53.237)	0.157
35–44	50	8	16.0 (5.84–26.16)	10.095 (1.214–83.930)	0.032
45–54	64	7	10.9 (3.29–18.58)	6.509 (0.775–54.683)	0.085
55–64	68	7	10.3 (3.07–17.52)	6.082 (0.725–51.044)	0.096
>65	47	2	4.3 (0.00–10.03)	2.356 (0.207–26.840)	0.490
**Region group**					**0.889**
Hunter valley/Central coast/Sydney	48	4	8.3 (0.51–16.15)	†	–
Regional NSW	268	24	9.0 (5.54–12.37)	0.924 (0.306–2.794)	0.889

*†Not applicable*.

### Seroprevalence of flaviviruses in rabbits

**Figure 3:**
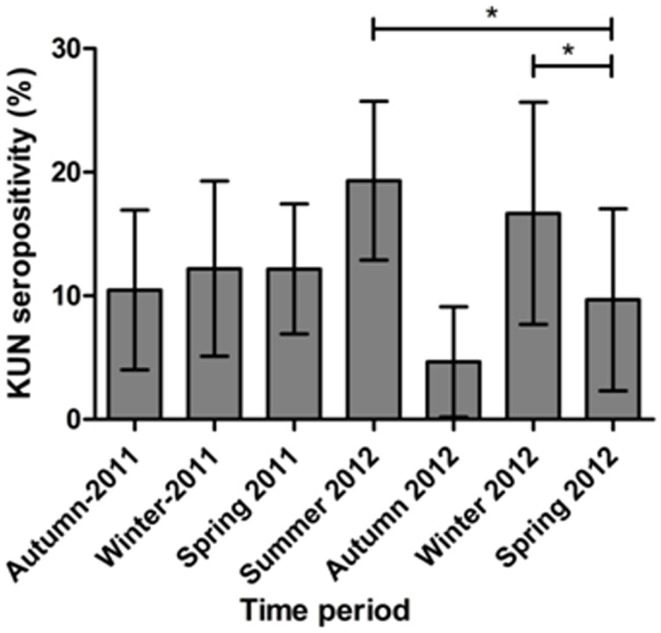
**WNV_KUN_ seroprevalence in feral rabbits in Australia sampled at the peak (Autumn 2011) and end (Winter 2011) of the equine encephalitis epidemic (Autumn 2011) and over the following 15 months**. Bars represent the proportion of animals seropositive at each time period, with the bars representing the 95% confidence intervals. Asterisks represent a significant difference (*p* < 0.05) compared to spring 2012.

Out of a total of 675 rabbits sampled between April 2011 (Southern Hemisphere Autumn) and November 2012 (Southern Hemisphere Spring) 86 animals (12.7%) had antibodies specific for WNV_KUN_ as determined in the 3.111G bELISA (Table 4). Of these, 28 (32.5% of bELISA positive) rabbits had WNV_KUN_ neutralizing antibodies with titers varying from 20 to 160, as determined in the microneutralization assay (data not shown). By univariate analysis, there was a significant association between state (*p* < 0.001) and time period (*p* = 0.050), while age had no apparent effect (*p* = 0.876). By multivariate logistic regression, both state (*p* < 0.001) and time period (*p* = 0.018) were still associated with seropositivity (Table 5). Specifically, the seroprevalence was higher in the states of Queensland and Victoria compared to Tasmania, and also in summer 2012 and winter 2012 compared to spring 2012 (Tables 4 and 5; Figures 2 and 3).

**Table 4:** **KUN seropositivity in Australian rabbits, from April 2011 to November 2012**.

	*n* Tested	KUN seropositive	
		*n*	% (95% CI)
Total	675	86	12.74 (10.23–15.26)
**TIME PERIOD**			
Autumn 2011	86	9	10.5 (4.00–16.93)
Winter 2011	82	10	12.2 (5.11–19.28)
Spring 2011	148	18	12.2 (6.90–17.43)
Summer 2012	145	28	19.3 (12.89–25.74)
Autumn 2012	86	4	4.6 (0.20–9.10)
Winter 2012	66	11	16.7 (7.68–25.66)
Spring 2012	62	6	9.7 (2.32–17.04)
**STATE**			
South Australia	109	4	3.7 (0.14–7.20)
Queensland	59	22	37.3 (24.95–49.63)
New South Wales	294	26	8.8 (5.60–12.09)
Northern Territory	60	7	11.7 (3.54–19.79)
Tasmania	19	1	5.3 (0.00–15.30)
Victoria	94	22	23.4 (14.84–31.96)
Western Australia	40	4	10.0 (0.70–19.30)
**AGE (MONTHS)**			
<3	53	8	15.1 (5.46–24.73)
3–5.9	84	7	8.3 (2.42–14.24)
6–8.9	108	14	13.0 (6.63–19.30)
9–11.9	79	9	11.4 (4.39–18.40)
12–14.9	56	8	14.3 (5.12–23.45)
≥15	278	37	13.3 (9.32–17.30)
N/A	17	3	–

**Table 5:** **Multivariate logistic regression analysis: influence of factors on KUN seropositivity in Australian rabbits**.

Variable	Multivariate logistic regression analysis		
	Odds ratio	95% CI	*p*-Value
**TIME PERIOD (REFERENCE GROUP: SPRING 2012)**			
Autumn 2011	0.554	0.178–1.721	0.307
Winter 2011	0.635	0.204–1.984	0.435
Spring 2011	0.608	0.219–1.688	0.340
Summer 2012	0.344	0.127–0.928	0.035
Autumn 2012	1.610	0.414–6.258	0.492
Winter 2012	0.275	0.090–0.842	0.024
**STATE (REFERENCE GROUP: TASMANIA)**			
South Australia	1.025	0.104–10.147	0.983
Queensland	0.060	0.007–0.500	0.009
New South Wales	0.363	0.044–2.975	0.345
Northern Territory	0.297	0.033–2.655	0.278
Victoria	0.105	0.013–0.868	0.037
Western Australia	0.256	0.026–2.559	0.246

## Discussion

West Nile virus is an on-going global public health concern, causing large outbreaks in the USA, Europe, and more recently Australia. Following extensive flooding across Eastern Australia in 2011 promoting ideal conditions for mosquito breeding, an unprecedented outbreak of equine encephalitis occurred, leading to the isolation of the first virulent strain of WNV in Australia. However, a number of unusual epidemiological features associated with this outbreak, including the lack of severe human cases, previously unseen transmission of WNV_KUN_ in coastal regions and a number of cases reported outside the areas affected by heavy rainfall, instigated further studies to try to explain the 2011 outbreak.

Historically, WNV_KUN_ has been associated with only mild symptoms in humans and horses. The emergence of the WNV_NSW2011_ strain suggested the virus may have undergone changes and evolved to become more virulent and able to cause fatal encephalitis in horses. However, since no severe human cases were seen during the outbreak, this study aimed to determine whether the lack of human involvement in the outbreak was simply due to pre-existing antibody against WNV_KUN_. The very low seroprevalence of WNV_KUN_ in humans observed in samples collected both before (0.7%) and after (0.6%) the 2011 outbreak of equine flavivirus encephalitis suggest that, in contrast to what was seen during the WNV incursion into the USA, humans residing in easternmost NSW were not exposed to the new WNV_NSW2011_. However, these seroprevalences are considerably lower than seen in previous serosurveys conducted in the Murray Valley region of NSW between 1991 and 2011, where WNV_KUN_ seroprevalences had been observed to range between 2.2 and 2.5% in the human population (21, 22). Higher WNV_KUN_ seroprevalence rates, ranging from 2.1 to 3.1%, have also been observed in Victorian residents (21, 23). It is possible that our results are biased because of the small sample size relative to previous studies and the fact that we studied a seemingly health conscious subset of the population, i.e., blood donors, who may take measures to limit their exposure to mosquitoes and thus arbovirus disease in accordance with Public Health recommendations. This suggestion is also supported by the absence of antibodies to MVEV despite 16 confirmed cases of MVEV in humans during 2011, as reported by the National Notifiable Diseases Network (12). However, it should be noted that the coastal regions of NSW are not typically affected by flavivirus diseases. Rather, MVEV tends to occur in the Murray Valley basin and occurrence is governed by rainfall and the resulting migration of birds. Previous VIC-based studies observed a MVEV seroprevalence of 3.7% (*n* = 2,783) in 1991–1992 and 2.2% (*n* = 1115) in 2011 (21, 23).

Another factor governing transmission of arboviruses is vector competence, which is the intrinsic ability for an arthropod vector to become infected with and transmit an arbovirus (24). Studies are currently being undertaken to determine whether the NSW2011 strain of WNV_KUN_ exhibits increased fitness in the primary vector, *Cx. annulirostris*, compared to non-pathogenic strains (van den Hurk et al., personal communication). If the NSW2011 strain is transmitted more efficiently by mosquitoes, then increased vector competence of *Cx. annulirostris* may have contributed to the equine epidemic, making it even more intriguing that corresponding increases in human exposures and clinical cases were not seen.

A large scale serological survey was conducted in the NSW equine population in late 2011 following cessation of the equine epidemic (25). This study had two notable findings, firstly that the overall seroprevalence of WNV_KUN_ in horses was low (3.9% of 1054 horses across the state) and lower than in previous studies. Secondly, almost all the seropositive horses came from far-western districts of NSW (25), while the majority of clinical cases were seen in the eastern half of NSW (14). This also suggests that infection with this new strain of WNV_KUN_ was associated with very high morbidity in the equine population.

In considering the distribution of equine encephalitis cases in the 2011 epidemic (14), we hypothesized that animal species other than birds might be involved in the spread of WNV_KUN_ along the eastern coastal areas of NSW. Notably, this area largely avoided the flooding events of early 2011 and dry conditions prevailed in the region during the summer and autumn of 2011. One species with wide distribution across Australia is the feral rabbit (www.invasiveanimals.com). Rabbits have been shown experimentally to develop a viremia in the absence of clinical signs following infection with WNV (16, 17). Interestingly, the present study revealed a higher seroprevalence in rabbits caught in Queensland and Victoria, compared to NSW. However, this finding may be biased due to the sampling areas, with the NSW sampling area being west of the main region for equine cases [(14); Figure 1]. In contrast, the high seroprevalence in Victorian rabbits is in concordance with the equine epidemic extending into this state, and the fact that the rabbit sampling area (Hattah-Kulkyne National Park) was within the region with many equine cases. The findings for Queensland are particularly notable, as this state avoided the equine encephalitis epidemic despite major flooding (14) and despite the presence of a susceptible equine population (20). The lack of correlation between equine clinical cases and seroprevalence in rabbits in some areas may be taken to suggest that rabbits have no role in the transmission cycle. Nevertheless, the finding that seroprevalence in this species was independent of age may suggest that they could be used as sentinels for arbovirus activity in a particular region. As the rabbit is a pest species in Australia and various programs are in place to control them, culled animals could be sampled for serological surveys prior to destruction and in this way inform Public Health authorities about potential arbovirus activity in a particular region.

WNV is estimated to have infected approximately four million humans in North America, causing over 37,000 clinical infections and 1443 deaths between 1999 and 2012 (4). On-going surveillance of currently circulating WNV strains in North America has indicated that the virus is continually changing with at least three different genotypes identified to date. Given that North American and Australian strains of WNV are very close genetically, sharing ~99% amino acid identity, and all WNV strains share a common transmission cycle, the possibility of emerging virulent strains of WNV in Australia, able to induce severe human disease, remains a definite possibility. In light of the observations from this study, the Australian population is still vulnerable to these emerging virulent strains, as very few people appear to have levels of WNV_KUN_-specific antibodies sufficient to afford protective immunity.

## Author Contributions

Conceived and designed the experiments: Natalie A. Prow, Helen M. Faddy, Roy A. Hall, Helle Bielefeldt-Ohmann. Performed the experiments: Natalie A. Prow, Elise K. Hewlett, Flaminia Coiacetto, Wenqi Wang, Tarnya Cox. Analyzed the data: Helen M. Faddy, Natalie A. Prow, Tarnya Cox, Helle Bielefeldt-Ohmann. Drafted the manuscript: Natalie A. Prow, Helen M. Faddy, Helle Bielefeldt-Ohmann. Revisions and final approval of the submitted version: all authors.

## Conflict of Interest Statement

The authors declare that the research was conducted in the absence of any commercial or financial relationships that could be construed as a potential conflict of interest.

## References

[1] Murgue B Zeller H Deubel V The ecology and epidemiology of West Nile virus in Africa, Europe and Asia. Curr Top Microbiol Immunol (2002) 267:195–221 12082990 10.1007/978-3-642-59403-8_10

[2] Lanciotti RS Roehrig JT Deubel V Smith J Parker M Steele K Origin of the West Nile virus responsible for an outbreak of encephalitis in the northeastern United States. Science (1999) 286:2333–7 10.1126/science.286.5448.2333 10600742

[3] Murray KO Mertens E Despres P West Nile virus and its emergence in the United States of America. Vet Res (2010) 41:67 10.1051/vetres/2010039 21188801 PMC2913730

[4] Petersen LR Brault AC Nasci RS West Nile virus: review of the literature. JAMA (2013) 310:308–15 10.1001/jama.2013.8042 23860989 PMC4563989

[5] Scherret JH Poidinger M Mackenzie JS Broom AK Deubel V Lipkin WI The relationships between West Nile and Kunjin viruses. Emerg Infect Dis (2001) 7:697–705 10.3201/eid0704.010418 11585535 PMC2631745

[6] Scherret JH Mackenzie J Hall RA Deubel V Gould EA Phylogeny and molecular epidemiology of West Nile and Kunjin Viruses. In: Mackenzie J Barrett AD Deubel V editors. Japanese Encephalitis and West Nile Viruses. New York: Springer-Verlag (2002). p. 373–90 10.1007/978-3-642-59403-8_18 12082998

[7] May FJ Davis CT Tesh RB Barrett AD Phylogeography of West Nile virus: from the cradle of evolution in Africa to Eurasia, Australia, and the Americas. J Virol (2011) 85:2964–74 10.1128/JVI.01963-10 21159871 PMC3067944

[8] Hall RA Broom AK Smith DW Mackenzie JS The ecology and epidemiology of Kunjin virus. Curr Top Microbiol Immunol (2002) 267:253–69 12082993 10.1007/978-3-642-59403-8_13

[9] Frost MJ Zhang J Edmonds JH Prow NA Gu X Davis R Characterization of virulent West Nile virus Kunjin strain, Australia, 2011. Emerg Infect Dis (2012) 18:792–800 10.3201/eid1805.111720 22516173 PMC3358055

[10] Ostlund EN Crom RL Pedersen DD Johnson DJ Williams WO Schmitt BJ Equine West Nile encephalitis, United States. Emerg Infect Dis (2001) 7:665–9 10.3201/eid0704.010412 11589171 PMC2631754

[11] Bunning ML Bowen RA Cropp CB Sullivan KG Davis BS Komar N Experimental infection of horses with West Nile virus. Emerg Infect Dis (2002) 8:380–6 10.3201/eid0804.010239 11971771 PMC3393377

[12] Knope K Whelan P Smith D Johansen C Moran R Doggett S Arboviral diseases and malaria in Australia, 2010-11: annual report of the National Arbovirus and Malaria Advisory Committee. Commun Dis Intell Q Rep (2013) 37:E1–20 23692155 10.33321/cdi.2013.37.1

[13] Prow NA The changing epidemiology of Kunjin virus in Australia. Int J Environ Res Public Health (2013) 10:6255–72 10.3390/ijerph10126255 24287851 PMC3881112

[14] Roche SE Wicks R Garner MG East IJ Paskin R Moloney BJ Descriptive overview of the 2011 epidemic of arboviral disease in horses in Australia. Aust Vet J (2013) 91:5–13 10.1111/avj.12018 23356366

[15] Kay BH Young PL Hall RA Fanning ID Experimental infection with Murray Valley encephalitis virus. Pigs, cattle, sheep, dogs, rabbits, macropods and chickens. Aust J Exp Biol Med Sci (1985) 63(Pt 1):109–26 10.1038/icb.1985.13 2990398

[16] Tiawsirisup S Platt KB Tucker BJ Rowley WA Eastern cottontail rabbits (*Sylvilagus floridanus*) develop West Nile virus viremias sufficient for infecting select mosquito species. Vector Borne Zoonotic Dis (2005) 5:342–50 10.1089/vbz.2005.5.342 16417430

[17] Suen W Prow NA Wang W Broad N Hall RA Kirkland PD The establishment of a rabbit model to elucidate mechanism of neuroinvasion by an emergent Australian West Nile virus. Queenstown: Australasian Virology Society Meeting (2013).

[18] Augusteyn RC On the relationship between rabbit age and lens dry weight: improved determination of the age of rabbits in the wild. Mol Vis (2007) 13:2030–4 17982428

[19] Hall RA Broom AK Hartnett AC Howard MJ Mackenzie JS Immunodominant epitopes on the NS1 protein of MVE and KUN viruses serve as targets for a blocking ELISA to detect virus-specific antibodies in sentinel animal serum. J Virol Methods (1995) 51:201–10 10.1016/0166-0934(94)00105-P 7738140

[20] Prow NA Tan CSE Wang W Hobson-Peters J Kidd L Barton A Natural exposure of horses to mosquito-borne flaviviruses in South-East Queensland, Australia. Int J Environ Res Public Health (2013) 10:4432–43 10.3390/ijerph10094432 24048209 PMC3799510

[21] Hawkes RA Pamplin J Boughton CR Naim HM Arbovirus infections of humans in high-risk areas of South-Eastern Australia: a continuing study. Med J Aust (1993) 159:159–62 8393128 10.5694/j.1326-5377.1993.tb137778.x

[22] Doyle JS Nicholson S Leydon JA Moran RJ Catton MG Opportunistic serological surveillance for Murray Valley encephalitis virus in Victoria, February-May 2011. Med J Aust (2012) 197:150 22860790 10.5694/mja12.10221

[23] Williams SA Richards JS Faddy HM Leydon J Moran R Nicholson S Low seroprevalence of Murray Valley encephalitis and Kunjin viruses in an opportunistic serosurvey, Victoria 2011. Aust N Z J Public Health (2013) 37:427–33 10.1111/1753-6405.12113 24090325

[24] Hardy JL Houk EJ Kramer LD Reeves WC Intrinsic factors affecting vector competence of mosquitoes for arboviruses. Annu Rev Entomol (1983) 28:229–62 10.1146/annurev.en.28.010183.001305 6131642

[25] Finlaison D Moloney BJ Kirkland PD A Serological Survey of Horses and Cattle in New South Wales in 2011 for Infection with Kunjin and Murray Valley Encephalitis Viruses. Sydney: Department of Agriculture, Fisheries and Forestry, NSW Government (2012).

